# HIV-associated Non-small-cell Lung Cancer with Rearrangement of the Anaplastic Lymphoma Kinase Gene: A Report of Two Patients

**DOI:** 10.7759/cureus.5466

**Published:** 2019-08-22

**Authors:** Rayli Pichardo, Rafael Francisco Go, Lang Qu, Lily Hussein, Shweta Gupta

**Affiliations:** 1 Internal Medicine, John H Stroger Jr. Hospital of Cook County, Chicago, USA; 2 Internal Medicine, West China School of Medicine, Sichuan University, Chengdu, CHN; 3 Hematology / Oncology, John H Stroger Jr. Hospital of Cook County, Chicago, USA; 4 Hematology / Oncology, John H Stroger, Jr. Hospital of Cook County, Chicago, USA

**Keywords:** non-small cell lung cancer, alk-positive adenocarcinoma, hiv infection

## Abstract

Non-small-cell lung cancer (NSCLC) is the most common non-AIDS defining cancer in patients infected with HIV and has the highest mortality rate among all cancers in this patient population. Treatment of NSCLC in HIV-positive patients is similar to that in HIV-negative patients, but less is known about the molecular characteristics of NSCLC in HIV-positive patients. This report describes two patients with HIV-associated NSCLC and rearrangements of the anaplastic lymphoma kinase (ALK) gene. The disease followed an indolent course in both patients.

## Introduction

Lung cancer is the leading cause of cancer-related mortality in the United States. Histologically, over 80% of lung cancers are non-small-cell lung cancers (NSCLCs), with approximately 3%-7% positive for anaplastic lymphoma kinase (ALK) gene rearrangements [1-2]. Targeted treatments have been extremely effective in patients with NSCLCs positive for ALK rearrangements [1-2]. Lung cancer is the most common non-AIDs defining cancer in patients infected with HIV [3]. Over one-third of all deaths in HIV-positive patients are cancer-related, with lung cancer deaths being the most common [1]. Although mutations of the epidermal growth factor receptor (EGFR) gene have been reported in lung cancers in HIV-infected patients, relatively little is known about other currently actionable molecular characteristics of lung cancer in this population [4]. This report describes two patients with HIV-associated NSCLCs positive for ALK rearrangements. To our knowledge, these are the first reported patients with these characteristics. A MEDLINE literature search via the Ovid platform did not reveal any similar reported cases as of June 2019.

## Case presentation

Case 1

**Figure 1:**
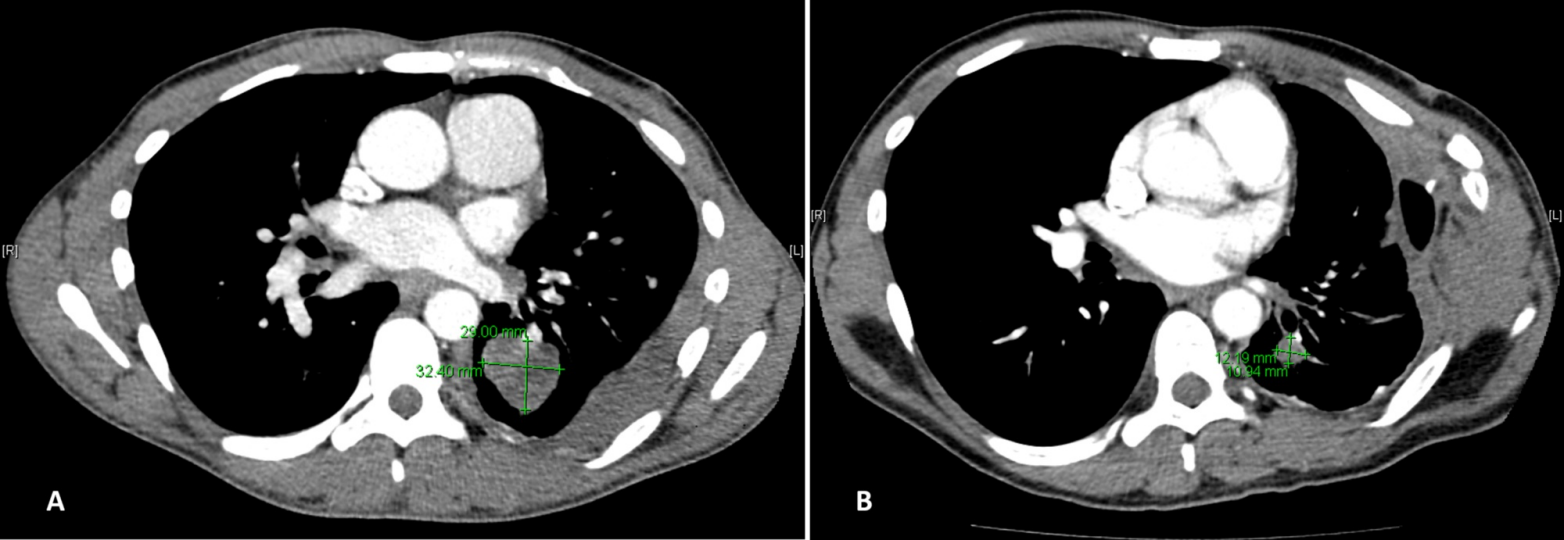
CT scans of the chest in Patient 1 in June 2017 (A) and June 2019 (B).

A 39-year-old African-American man, infected with HIV since 2008 and having a five-pack/year history of smoking, was incidentally found in April 2014, at age 34 years, to have a left lower lobe pulmonary nodule, 2.7 cm in diameter, during follow-up after an abnormal chest X-ray. CT scans of head, chest, abdomen, and pelvis did not show any other lesions. His CD4 count at diagnosis was 256 cells/mL, with an undetectable HIV viral load. A biopsy confirmed NSCLC (adenocarcinoma), but the patient refused treatment and was lost to follow-up. At this stage mutational analysis was not performed due to localized disease. In March 2015, he presented with shortness of breath and was found to have worsening lung disease with new pleural nodules. At that time, he was receiving highly active anti-retroviral therapy (HAART), consisting of tenofovir disoproxil fumarate-emtricitabine and dolutegravir. A left thoracotomy with parietal pleural biopsy confirmed NSCLC (adenocarcinoma). Fluorescence in-situ hybridization analysis showed that the lung nodules were positive for ALK rearrangement but negative for mutations of the EGFR and Kristen rat sarcoma (KRAS) mutations. His CD4 count was 342 cells/mL, and his HIV viral load was undetectable. He was started on oral crizotinib 250 mg twice per day. His HAART was switched to tenofovir alafenamide-emtricitabine with dolutegravir prophylactically due to renal toxicity known to be associated with both tenofovir disoproxil fumarate and crizotinib. He took crizotinib for one month but did not follow up with oncology because he again felt asymptomatic and refused further treatment. He continued to follow with the HIV clinic and remained compliant with HAART. In June 2017, he presented with worsening shortness of breath. A chest CT scan revealed significant progression of the disease, with a left lower lobe lung mass, left pleural nodules, left axillary, bilateral hilar lymphadenopathy, and multiple pulmonary nodules (Figure 1A). A brain MRI scan showed two 5 mm metastatic lesions in the right cerebellum, one 4 mm lesion each in right inferior parietal lobe and right temporal lobe. He underwent whole-brain radiation therapy (30 Gy in 10 fractions) and was restarted on crizotinib in July 2017. His response after three months was good with decrease in overall size and number of the lung lesions (largest lung lesion decreased from 3.3 cm to 2.6 cm) with stable brain lesions on MRI. Alectinib was FDA-approved in the front line setting four months later, however, it was decided to continue crizotinib as he was responding. In May 2018, he developed a large left-sided malignant pleural effusion with a progressive collapse of the left lower lobe and a partial collapse of the left upper lobe. A brain MRI showed two new lesions 2 mm each in the right and left frontal gyrus. His CD4 count at the time of progression was 308 cells/mL. He was switched to oral alectinib 600 mg twice daily, which he tolerated well, and the disease responded with significant decrease in pleural effusion and decrease in size of the lung nodules. As of June 2019 (5.2 years from initial diagnosis), he is compliant with therapy and continues to be asymptomatic with stable radiologic disease (Figure 1B). 

Case 2

**Figure 2:**
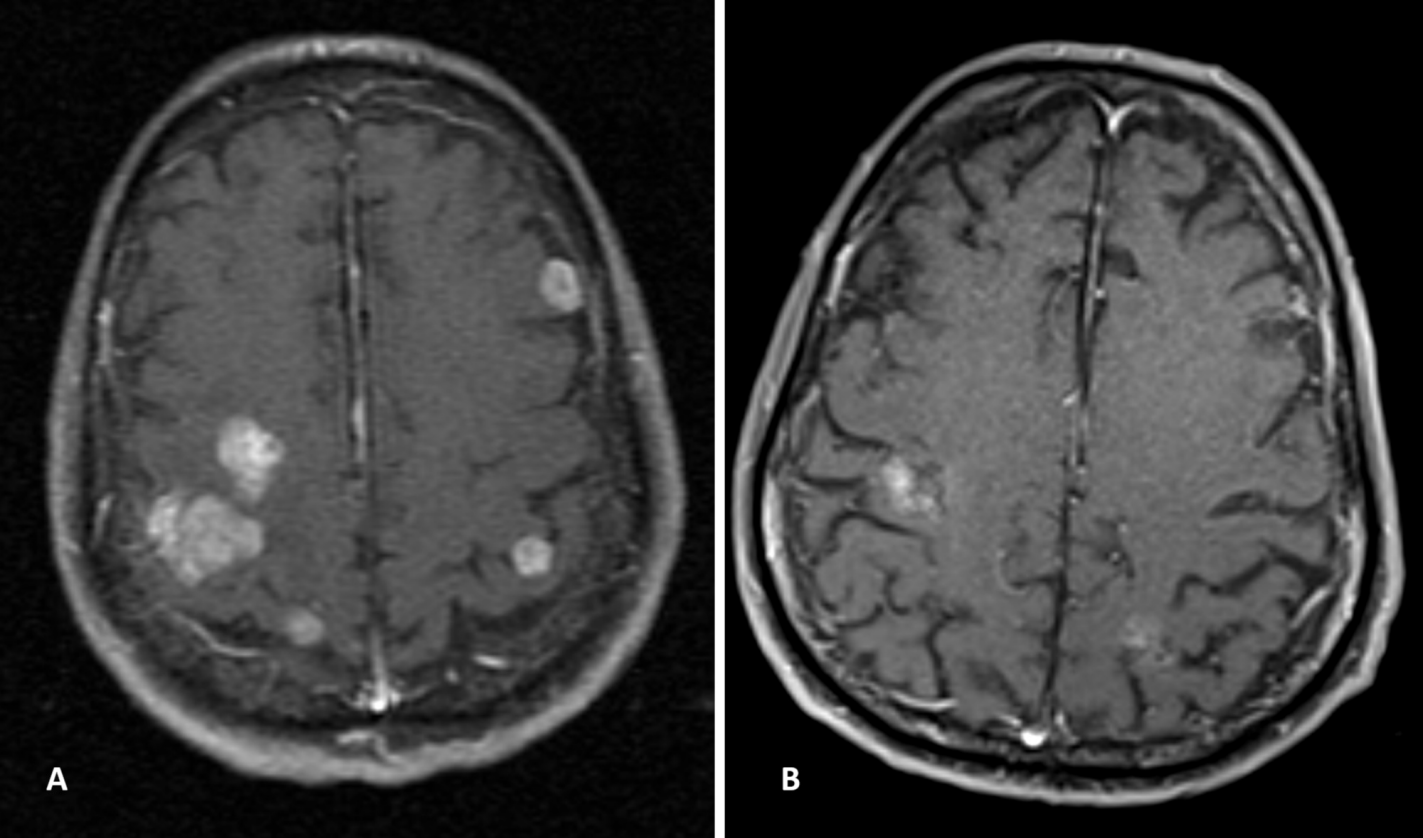
MRI scans of the brain in Patient 2 in February 2017 (A) and January 2019 (B).

A 65-year-old African-American man, nonsmoker, presented with dry cough in December 2011, at age 57 years. He was diagnosed with HIV in 2000 and had hypertension and coronary artery disease, for which he underwent coronary artery bypass grafting in 2003. He also had dyslipidemia, untreated hepatitis C virus infection, and history of coccidioidomycoses meningitis. A CT scan showed a 2.6 cm right lower lobe mass and a small sub-centimeter left upper lobe lung nodule. A whole body positron emission tomographic (PET) scan showed isolated uptake in the right lower lobe mass. His CD4 count at presentation was 162 cells/mL with undetectable viral load. He was being treated with HAART, consisting of raltegravir, etravirine, lamivudine, darunavir, and ritonavir. He underwent resection of his right middle and lower lobes with level 10 sampling of four lymph nodes. Pathologic examination of the tumor showed a poorly differentiated adenocarcinoma (stage T3N1). Because of his multiple medical co-morbidities, and after careful discussions with an infectious disease (ID) specialist and the patient, adjuvant therapy was withheld, and the patient was monitored closely. In March 2013, the left upper lobe nodule increased in size to 11 mm. He underwent video-assisted thoracic surgery with left wedge resection; pathologic examination of the tissue specimens confirmed poorly differentiated adenocarcinoma. An examination in December 2013 showed progressive development of right paratracheal and subcarinal lymphadenopathy, suggesting tumor progression. The previous pathology specimen tested positive for ALK rearrangement and negative for EGFR and KRAS mutations. In January 2014, he was started on oral crizotinib 250 mg twice daily and was switched from the antiretroviral medications ritonavir and darunavir to enfuvirtide prophylactically because ritonavir and darunavir have strong CYP34A inhibitor activity and may increase serum crizotinib levels. He responded well for three years but developed left arm weakness in February 2017 with MRI showing multiple new brain metastases (Figure 2A). Correspondingly no disease was detected on a chest CT scan. He was administered whole-brain radiation therapy (30 Gy in 10 fractions) and switched to ceritinib, which he was unable to tolerate due to intolerable diarrhea. In March 2017, he was switched to oral alectinib 600 mg twice daily, which he continues to tolerate well as of June 2019. His chest disease remains in remission, and his brain lesions have shown significant improvement with several lesions becoming inconspicuous (Figure 2B). He continues to do well, 7.5 years after initial presentation.

## Discussion

Lung cancer is the most common non-AIDS defining cancer affecting HIV-infected patients, independent of exposure to tobacco. HIV-infected patients are younger than noninfected patients at diagnosis of lung cancer. The introduction of combination antiretroviral treatment (cART) has resulted in significant reductions in mortality from HIV, leading to longer life expectancy in these patients. Longer life expectancy, however, is accompanied by higher co-morbidity rates, including higher rates of cancer. Although HIV infected patients with cancer had a very poor prognosis, the introduction of cART has improved their prognosis, although it remains inferior to cancer in uninfected patients [1, 5].

The molecular characteristics of HIV-associated lung cancer and differences between infected and uninfected patients remain unclear. Genomic alterations of microsatellite instability have been observed frequently in HIV-associated lung carcinomas, but the mechanism underlying the development of these alterations and their effects on lung cancer are not fully understood [4]. Although the occurrence of clinically relevant oncogenic mutations, including EGFR mutations in NSCLCs, was reported similar in HIV-infected and uninfected patients [6], to our knowledge, ALK rearrangements have not been previously reported in patients with HIV-associated lung cancer. 

Treatment with ALK inhibitors was shown to significantly improve the overall survival and progression-free survival of NSCLC patients positive for ALK rearrangements or ROS1 gene fusion. In non-HIV population, first-generation ALK inhibitors, such as crizotinib, had overall response rates (ORRs) of 57%-74%, whereas the second-generation ALK inhibitor alectinib had ORRs >80% in treatment-naïve populations. In comparison, chemotherapy alone had an ORR of 20% in the same population. Alectinib was also found to have a good intracranial response with ORRs of 40%-57% [2, 7]. However, response to ALK inhibitors has not been evaluated in HIV-positive patients because no such patient has been reported. 

Both our patients had an indolent course with good responses to initial crizotinib and further continued responses to alectinib after progression. Both patients had brain metastases, with tumors remaining controlled in these patients with alectinib over five and seven years, respectively, after initial cancer diagnosis. Both of these patients, however, required modification of HAART regimens when started on crizotinib, with no reported adverse effects from either crizotinib or alectinib. In contrast, ceritinib caused intolerable GI side effects in one patient.

Crizotinib and ceritinib are metabolized by CYP3A4 and are substrates of the efflux transporter P-glycoprotein. They can cause QT prolongation and significant interactions can occur if combined with other CYP3A4 inhibitors. Use of strong CYP3A4 inhibitors should be avoided with these agents [8-9]. Alectinib is metabolized by the CYP3A4 pathway into its active metabolite M4. However, other than enhancing the bradycardic effect of bradycardia-causing medications, no significant drug interactions have been reported between alectinib and other CYP3A4 inducers, possibly making it a safer choice [10].

## Conclusions

The similar prevalence of EGFR mutations in HIV-infected and uninfected patients with lung cancer and the good response to ALK inhibitors in our HIV-infected NSCLC patients suggest that all patients with HIV-associated NSCLC, especially adenocarcinomas, and no or light smoking history, should be screened for underlying molecular alterations such as EGFR and ROS1 mutations and ALK translocations. Screening of genetic alterations in these patients may allow the most appropriate treatment, as well as furthering understanding of the prevalence of molecular abnormalities in HIV-associated NSCLC patients. However, it is imperative to determine possible interactions between cART therapy and targeted ALK inhibitors and for teams of ID specialists, oncologists, and ID pharmacists to manage these patients together, thus avoiding drug-drug interactions and reducing the risk of adverse effects. 
